# Bioactive Compounds and Antioxidant Capacity in Pearling Fractions of Hulled, Partially Hull-Less and Hull-Less Food Barley Genotypes

**DOI:** 10.3390/foods10030565

**Published:** 2021-03-09

**Authors:** Mariona Martínez-Subirà, María-Paz Romero, Alba Macià, Eva Puig, Ignacio Romagosa, Marian Moralejo

**Affiliations:** AGROTECNIO-CERCA Center, University of Lleida, Av. Rovira Roure 191, 25198 Lleida, Spain; mariona.martinez@udl.cat (M.M.-S.); mariapaz.romero@udl.cat (M.-P.R.); alba.macia@udl.cat (A.M.); eva.puig@udl.cat (E.P.); ignacio.romagosa@udl.cat (I.R.)

**Keywords:** whole barley flour, pearling fractions, proteins, β-glucans, arabinoxylans, tocols, phenolic compounds, antioxidant capacity, functional food

## Abstract

Three food barley genotypes differing in the presence or absence of husks were sequentially pearled and their fractions analyzed for ash, proteins, bioactive compounds and antioxidant capacity in order to identify potential functional food ingredients. Husks were high in ash, arabinoxylans, procyanidin B3, prodelphinidin B4 and *p*-coumaric, ferulic and diferulic bound acids, resulting in a high antioxidant capacity. The outermost layers provided a similar content of those bioactive compounds and antioxidant capacity that were high in husks, and also an elevated content of tocols, representing the most valuable source of bioactive compounds. Intermediate layers provided high protein content, β-glucans, tocopherols and such phenolic compounds as catechins and bound hydroxybenzoic acid. The endosperm had very high β-glucan content and relative high levels of catechins and hydroxybenzoic acid. Based on the spatial distribution of the bioactive compounds, the outermost 30% pearling fractions seem the best option to exploit the antioxidant capacity of barley to the full, whereas pearled grains supply β-glucans enriched flours. Current regulations require elimination of inedible husks from human foods. However, due to their high content in bioactive compounds and antioxidant capacity, they should be considered as a valuable material, at least for animal feeds.

## 1. Introduction

Barley (*Hordeum vulgare* L.) is the fourth most cultivated cereal in the world after maize, wheat and rice [1]. It is widely used for feed and malt, with limited consumption as human food in some specific regions, such as the Maghreb and the high plateaus of the Himalayas. However, in recent years, it has attracted growing interest worldwide due to the health promoting properties of its bioactive compounds. In terms of health, several reports have demonstrated the positive effect of barley on the glycemic index, cholesterol and heart diseases [2]. This is mainly due to the presence of β-glucans (2–11% d.w.), a dietary fiber component for which health claims have been approved by the US Food and Drug Administration and the European Food Safety Authority [3,4]. Additional beneficial effects have been described for β-glucans. These include their properties as enhancers of the immune system against infectious diseases and some types of cancer, and also as a key modulator of the composition and activity of human microbiota [5,6,7]. Arabinoxylans are the second major component of barley dietary fiber (2–9% d.w.). Their proven health benefits include effects on the postprandial glucose response, cholesterol metabolism and immune response [8,9]. In barley, arabinoxylans are particularly associated with antioxidant activity due to the presence of phenolic acids linked to their structure [10]. Barley is also a significant source of antioxidant compounds [11,12]. It contains more vitamin E than most cereals (17–49 μg/g d.w.), this being in the form of α-tocopherol and α-tocotrienol. Furthermore, barley contains high levels of phenolic compounds such as phenolic acids and flavan 3-ols which can be found free or ester-bound to the fiber. Flavan 3-ols like catechins, procyanidins and prodelphinidins are the most abundant free phenols, while phenolic acids such as ferulic, *p*-coumaric and vanillic are the major constituents within the bounds. Anthocyanins are also present in considerable concentrations in some barley genotypes with colored kernels. All these phenolic compounds are considered potent antioxidants, free radical scavengers and inhibitors of lipid peroxidation [13,14]. In addition, preclinical studies and clinical trials have shown that polyphenols could greatly modulate the gut microbiota, thus favoring the growth of potential beneficial organisms and simultaneously inhibiting pathogenic bacteria [15].

Barley grains may differ in important morphological characters. These include being hulled (covered), when the husks adhere to the caryopsis, partially hull-less (skinned), when a partial loss of the husks occurs, and hull-less (naked), when the husks are freely threshed at harvest, the latter being what is preferred for human consumption; grains from two or six rowed spikes or grains with colors such black, blue or purple, alternative to yellow. Based on the grain composition, barley is further classified as normal, high amylopectin (waxy) or high amylose starch types, high β-glucan, high lysine, and proanthocyanidin-free. All these types of grain differ widely in their physical and compositional characteristics and, accordingly, are processed differently and used for different commercial purposes.

The bioactive compounds are not uniformly distributed across the barley grains. It is known that arabinoxylans, tocotrienols and phenolics are mainly located in the outer layers, tocopherols in the germ, and β-glucans in the endosperm [16]. Thus, physical processes like pearling, an abrasive technique that gradually removes grain layers to obtain polished grain and by-products, allow favorable separation of fractions enriched in specific compounds and these can be used as functional ingredients. Several fold enrichment of antioxidant compounds has been described in barley pearled fractions [17,18] that have been used to improve the nutritional value of such wheat-based products as cookies, pasta and bread [19,20,21,22].

In recent years, increased efforts have been carried in a few countries to release new specific barley varieties for human consumption and for the food industry. In our food barley breeding program, we aim to produce varieties rich in β-glucans as well as antioxidant compounds adapted to the Spanish agro-climatic conditions. Three distinct high β-glucan barley genotypes from our program, differing in the type of grain (Kamalamai, hulled; Hindukusch, partially hull-less; Annapurna, hull-less) were selected to identify specific potential ingredients for the functional food industry. Thus, the main objectives of this work were as follows: (1) to analyze β-glucans, arabinoxylans, tocols, phenolic compounds and antioxidant capacity in the whole flours and pearling fractions of different types of barley grains; (2) to identify pearling fractions to be used potentially as functional ingredients. This research could provide further knowledge about the spatial distribution of a large number of bioactive compounds in barley pearling fractions, since most published works have focused on changes in various bioactive compounds separately [16,17,23]. To the best of our knowledge, this is the first time that such an integrative study of the major health-promoting components in barley genotypes specifically bred for human food has been explored.

## 2. Materials and Methods

### 2.1. Plant Material

- Kamalamai: registered Spanish variety, hulled, two rowed, normal endosperm (semillas Batlle SA).
- Hindukusch: Afghan landrace, naked but often suffering from grain skinning, two rowed, normal endosperm and purple grain used as parent in our crosses.
- Annapurna: registered Spanish variety, hull-less, two rowed, waxy endosperm (semillas Batlle SA).

The three genotypes were cultivated under similar conditions in Bell-lloc d’Urgell, Lleida (Spain) during the 2018–2019 growing season.

### 2.2. Whole Flours and Pearling Fractions

Grain with size above 2.5 mm was screened for this study using a stainless-steel mesh. Six pearling fractions from each genotype were obtained by sequential processing of grain using theTM-05C pearling machine (Satake Corporation, Hiroshima, Japan) at 1060 rpm. The grains were initially pearled to remove the 5% of the original grain weight that resulted in the first fraction F1 (0–5% *w/w*). The remained grains were pearled to remove the second fraction F2 (5–10%), and then the process was repeated to get fractions F3 (10–15%), F4 (15–20%), F5 (20–25%), F6 (25–30%), and the residual 70% pearled grain F7 (30–100% *w/w*). After each pearling session, the pearling machine was cleaned to avoid mixtures between fractions. Fractions, pearled grains and whole grains were ground in a Foss Cyclotec 1093™ mill equipped with a 0.5-mm screen (Foss Iberia, Barcelona, Spain) prior to chemical analyses.

### 2.3. Protein and Ash Content

The protein content was done according to the Kjeldahl method in a Kjeltec system I (Foss Tecator AB, Höganäs, Sweden) using the conversion factor of 5.7. The ash content was determined in a muffle furnace according to the AOAC Official Method 942.05 [24].

### 2.4. β-Glucan and Arabinoxylan Content

The β-glucan and arabinoxylan contents were determined by means of the mixed-linkage β-glucan assay (K-BGLU) and D-xylose assay (K-XYLOSE) kits from Megazyme (Wicklow, Ireland).

### 2.5. Tocols Content

Tocopherols and tocotrienols (α-, β-, γ-, and δ-isomers) were quantified by high performance liquid chromatography (HPLC) coupled to a fluorescence detector. One gram of each barley genotype was extracted three times with 10 mL n-hexane and the extract collected after centrifuging at 9000× *g* for 10 min. The supernatants were pooled, reduced to dryness under a flow of nitrogen, and reconstituted in 1 mL of n-hexane. Normal phase HPLC with fluorescence detection (excitation 292 nm, emission 325 nm) was used to analyze tocopherols and tocotrienols. Aliquots of 50 μL were injected into the HPLC system following the chromatographic conditions described by Martínez-Subirà et al. [12]. Tocopherols and tocotrienols isomers were quantified with external standard curves. Results were expressed as μg/g dry sample.

### 2.6. Phenolic Compounds (PCs) and Anthocyanin Analysis by UPLC-MS/MS

Free phenolic compounds were extracted three times by adding 1 mL of 79.5% methanol, 19.5% Milli Q water, and 1% formic acid solution to 150 mg of barley flours. The samples were sonicated for 30 s and centrifuged at 9000× *g* for 10 min. The supernatants from each extraction were pooled and filtered through 0.22 μm polyvinylidene fluoride (PVDF) filter discs before chromatographic analysis. The residue was subjected to alkaline hydrolysis by adding 6 mL of 2 mol/L NaOH to obtain bound phenols. The samples were left over-night at room temperature for complete hydrolysis. Then, they were sonicated for 1 min and centrifuged at 9000× *g* for 10 min; the supernatant was acidified with HCl 37% (*w/w*) to pH 2. A total of 350 μL of supernatant was mixed with phosphoric acid 10 min, centrifuged at 9000× *g*, and subjected to μSPE clean-up according to Serra et al. [25]. Briefly, the micro-cartridges were pre-conditioned with acidified water (pH 2) and methanol. The samples were loaded onto the μSPE and subsequently washed with water and water/methanol 95/5 (*v/v*). The PCs were eluted with methanol, and 2.5 μL of the eluate was directly analyzed by liquid chromatography. The extracts were analyzed by Ac Quity Ultra-Performance ^TM^ liquid chromatography coupled to a tandem mass spectrometer (UPLC-MS/MS), equipped with the analytical column Ac Quity BEH C18 (100 mm × 2.1 mm i.d., 1.7 μm) and the Van Guard TM Pre-Column Ac Quity BEH C 18 (5 mm × 2.1 mm, 1.7 μm), all from Waters, Milford, MA, USA. The mobile phase was 0.2% (*v/v*) acetic acid and acetonitrile for the phenolic compounds (PCs), and 10% acetic acid (*v/v*) and acetonitrile for the anthocyanins. The UPLC system was coupled to a triple quadrupole detector mass spectrometer from Waters equipped with a Z-spray electrospray interface for ionization, operating in the negative mode [M−H]^−^ for the PCs and the positive mode [M−H]^+^ for the anthocyanins. Quantification was based on a 0.02–25 ng calibration curve of commercially available standards, and the results were expressed as μg/g dry sample. A linear response was obtained for all standards and tested by linear regression analysis. The limits of detection (LOD) ranged from 0.007 to 0.09 ng and the limits of quantification (LOQ) from 0.02 to 0.30 ng.

### 2.7. Antioxidant Capacity (AC) Analysis

The Oxygen Radical Absorbance Capacity (ORAC) of both the free and bound extracts was measured as described Huang et al. [26]. The antioxidant capacity (AC) was determined using the FLUO star OPTIMA fluorescence reader (BMG Labtech, Offenburg, Germany) in a 96-well polystyrene microplate controlled by the OPTIMA 2.10 R2 software. Changes in fluorescence were measured under controlled temperature (37 °C) in a reader with fluorescence filters with 485 nm excitation and 520 nm emission wavelengths. Trolox (6-hydroxy-2, 5, 7, 8-tetramethylchroman-2-carboxylic acid) was used as control, with one ORAC unit being equal to the antioxidant protection given by 1 μmol Trolox. The results were expressed as μmols of Trolox-equivalents per 100 g of dry sample.

### 2.8. Statistical Analysis

All statistical analyses were conducted using JMP^®^Pro14 (SAS institute Inc., Cary, NC, USA). Analytical measurements were carried out in triplicate and the results were presented as mean values. Data was checked for normality and for homoscedasticity of variances based on a number of diagnostic tools, such as residual plots, Box-Cox′s Lambda and Levene’s test of equality of variances, provided by JMP. When needed, a logarithmic transformation was used and indicated in the text. For multiple comparisons, Tukey–Kramer’s honestly-significant-difference tests (HSD) (α = 0.05) were conducted once the corresponding ANOVA F-tests were found significant. Principal Component Analysis (PCA) was used to graphically represent the association between fractions, genotypes and bioactive compounds using standardized data.

## 3. Results and Discussion

### 3.1. Ash and Protein Contents

The ash and protein contents of the whole flours and pearling fractions are shown in Table 1. The hulled genotype Kamalamai showed the highest ash content and Hindukush, the partially hull-less genotype, the highest protein amount. The distribution through the grain showed a progressive decrease in the ash content from the first pearling fraction F1 of the three genotypes toward the endosperm. This was because the mineral components are mainly localized in the outer layers of the kernel. The protein content was the lowest in the initial surface F1 fraction of the Kamalamai, which mainly correspond to husks, whereas the highest content was detected in the middle fraction F4 of both genotypes containing husks, and F3 of the hull-less Annapurna genotype. These results were in accordance to that observed by other authors on barley fractions obtained by pearling or roller-milling processes [16,22].

### 3.2. β-Glucans, Arabinoxylans and Tocols Contents

**Figure 1:**
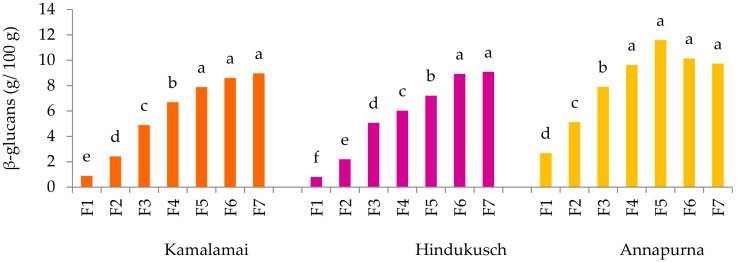
β-Glucan content (g/100 g) in barley pearling fractions. The results are presented as the mean; different letters indicate significant differences within the pearled fraction of each genotype; (Tukey-Kramer’s HSD for α = 0.05). F1–7: fraction1–7.

In barley, the β-glucan content depends on genetic and environmental factors as well as on the interaction between these [8,27]. In our work, the three barley genotypes were high in β-glucans with amounts ranging from 8.3 to 9.5 g/100 g (Table 2). Annapurna had the highest β-glucan content while both genotypes containing husks showed similar values. These results agree with earlier studies which described higher β-glucan levels in hull-less and waxy genotypes like Annapurna rather than in hulled barley with normal endosperm [2,28]. The distribution of this fiber component through the grain is shown in Figure 1. β-glucans increased gradually from the outer to inner layers of the grains in accordance with previous findings [22,29]. The lowest concentrations were detected in the outer fraction F1 of the three genotypes and progressively increased until F4 of Annapurna, F5 of Kamalamai, and F6 of Hindukusch, after wich they remained constant. The net effect was that removing the 30% outer fractions of Kamalamai and Hindukusch increased the β-glucan content by 9% and 6%, respectively, while this increase was only 3% in the hull-less genotype Annapurna.

**Figure 2:**
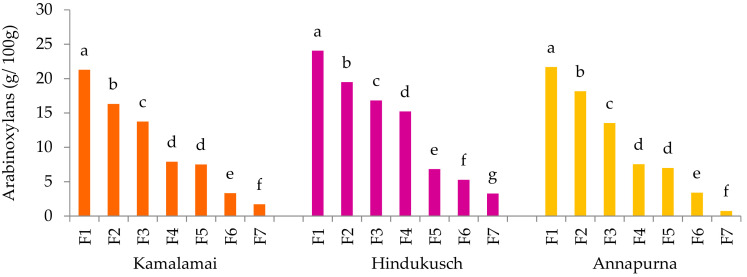
Arabinoxylan contents (g/100 g) in barley pearling fractions. Results are presented as the mean; different letters indicate significant differences within pearled fraction of each genotype; (Tukey-Kramer’s HSD for α = 0.05).

Arabinoxylans in cereals are mainly localized in the husks and cell walls of the outer layers of the grain including pericarp, testa and aleurone. In barley, aleurone cell walls are built up mainly of arabinoxylans (60 to 70%) whereas the endosperm cell walls contain only about 20 to 40% [30]. In our work, the arabinoxylan content of whole barley flours ranged from 5.5 to 6.6 g/100 g; Hindukusch being the highest (Table 2). These values were in accordance with those reported for different barley accessions in a previous work [31]. Contrary to the β-glucans, the arabinoxylans decreased progressively from the outer to the inner layers of the grain (Figure 2). The highest arabinoxylans amounts were observed in all F1 fractions. These mainly correspond to the hulls, testa and to the pericarp of both genotypes containing husks, and to pericarp, testa and some aleurone layers of Annapurna. The arabinoxylan level detected in the outermost layer F1 of the three genotypes was on average four times higher than that in whole barley flours. This finding may draw attention to barley husks as a good source of arabinoxylans.

**Figure 3:**
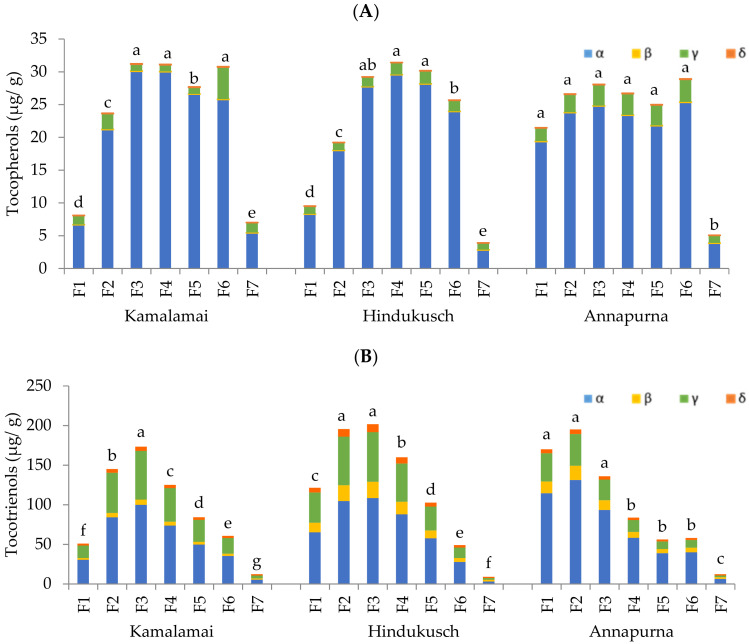
(**A**) α-, β-, γ-, δ- Tocopherol contents (µg/g), and (**B**) α-, β-, γ-, δ- Tocotrienol contents (µg/g) in barley pearling fractions. Results are presented as the mean; different letters indicate significant differences between pearled fractions of each genotype on log-transformed data; (Tukey-Kramer’s HSD for α = 0.05).

Tocols are plant metabolites with interest for their potential benefits for human health [32]. Tocols, which consist of tocopherol and tocotrienols, are found in cereals at moderate levels ranging between 17 and 49 μg/g [17,33]. Among cereals, barley is one of the best sources of tocols due to the high content and favorable distribution of all eight major tocols, α-, β-, γ- and δ- tocopherols and tocotrienols [34]. While all tocol forms have similar antioxidant properties, α tocopherol (αT) is the only one that meets the Recommended Daily Allowance for vitamin E [35,36]. In our study, there were detectable concentrations of the eight tocol isomers in all whole barley flours (Table 2). Total tocols ranged from 38 to 44 μg/g in good accordance with values found in other barley cultivars [12,16,17,18]. Tocotrienols accounted for 79% of the total tocols while the tocopherols were 21%. αT and α tocotrienols (αT3) were the main isomers in each tocol class, and their concentrations ranged from 6.2 to 8.3 μg/g and 17 to 20 μg/g respectively. Significant differences were identified among genotypes for most tocol forms. Both genotypes containing husks were the highest in αT, γT3 and total tocols. The tocopherol and tocotrienol contents in the fractions are shown in Figure 3A,B and Table S1. In line with previous finding, tocopherols and tocotrienols are distributed in a tissue specific manner with tocopherols mainly located in the germ whit tocotrienols in the aleurone and sub-aleurone layers [16,17,35]. In this study, the highest tocopherol contents were detected between the middle fractions F3 to F6 of genotypes containing husk. This correspond to the highest amount of protein as described above, whereas the content was uniformly distributed across the grain in the hull-less genotype Annapurna. Tocotrienols were more abundant in the outer layers F2 and F3 of the genotypes containing husks, and F1 to F3 of the hull-less one. Fractions F2 to F4 of the genotypes containing husks, and F1 to F3 of Annapurna would provide average tocol contents of 194 μg/g. These selected fractions contain over five times more tocols than whole flours and could be used as a valuable source of natural tocols.

### 3.3. Phenolic Compounds (PCs) Contents

Phenolic compounds in barley can be found free or bound to the fiber being differentially distributed across the grain [37]. The analysis of PCs in whole barley flours carried out by ultra-performance liquid chromatography-tandem mass spectrometer (UPLC-MS/MS) included free and bound forms (Table 3). Total free PCs ranged from 369 to 600 μg/g among which, flavan-3-ols accounted for 80%, phenolic acids for 16% and flavone glycosides for 4.1%. Total bound PCs ranged from 781 to 1194 μg/g and were comprised of 99.8% of phenolic acids and 0.2% of flavone glycosides. Bound phenolic acids were predominant in all whole flours representing 65–76% of the total PCs.

Looking at the pearling fractions, the results showed a wide range of phenolic contents with differences between fractions and genotypes. Twenty-two different free phenols were detected. Their distribution within the fractions is detailed in Table 4. Procyanidin B3 and Prodelphinidin B4 were the most abundant flavan-3-ols in all the external fractions. Fractions F2 and F3 of the genotypes containing husks and F1 and F2 of Annapurna showed the highest concentrations of most flavan-3-ols which then progressively decreased until the endosperm except for catechins whose content did not vary as much between fractions. Fraction F1 was the richest in free phenolic acids in the three genotypes. This excludes gallic acid (the major free phenolic acid) whose content was similar within the external fractions. Some free phenolic acids, such as decarboxylated diferulic, hidroxybenzoic, caffeic and cinnamic, were exclusively detected in F1, F2 and F3 and were absent in the rest of the grain. Moreover, decarboxylated diferulic, hydroxybenzoic and 2,4-dihydroxybenzoic were not detected in Annapurna. F1 and F2 had also the highest concentrations of some free flavone glycosides such as apigenin 6-C-arabinoside 8-C-glucoside and ixovitexin 7-rutinoside, while the concentration of the remained free flavones was homogeneous or randomly distributed across the grain. Isoorientin was not detected in Annapurna nor was isovitexin 7-(6’’’-sinapoylglucoside) 4’-glucoside in Hindukusch and Annapurna.

With reference to bound phenolic acids, their distribution across the grain was similar in the three genotypes (Table 5). The highest values were detected in the outermost fraction (F1) and gradually decreased toward the core of the grain, as did arabinoxylans, with which they are esterified. In fact, arabinoxylans positively correlated with bound phenolic acids (*r* = 0.93, *p* < 0.001). Ferulic acid was found to be the most abundant bound phenolic acid found in the three genotypes, accounting for 68–83% of total bound; p-coumaric acid came second in the genotypes containing husks (12–15%) and the diferulic acid in Annapurna (6%). Several authors detected high amounts of bound phenolic acids in husks [29,30]. This might explain the results observed in our study: the genotypes containing husks being the richest in these compounds. In general, F1 was remarkably high in bound phenolic acids and might provide an important source of antioxidants. Minor bound phenolic acids, such as hydroxybenzoic acid, were uniformly distributed across the grain, and the distribution of others, like isoferulic, hydroxybenzoic, 2,4-dihydroxybenzoic, caffeic, sinapic and cinnamic acids, was not very marked. Bound diferulic tetrahydrofuran acid seemed to be exclusively present in the husks and pericarp layers as it was only detected in the F1 and F2 of both genotypes containing husks. Bound flavone glycosides showed similar distribution pattern as did the free forms.

**Figure 4:**
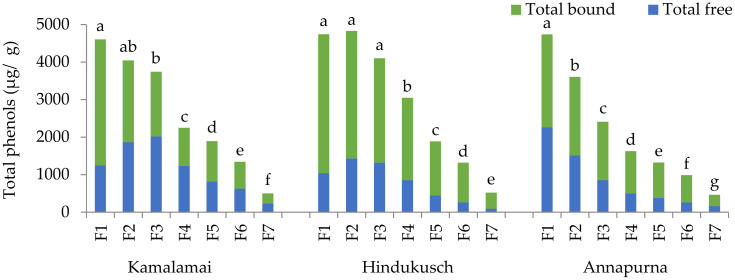
Total phenols contents (µg/g) in seven pearling fractions of three food barley genotypes. Results are presented as the mean; different letters indicate significant differences between pearled fractions of each genotype on log-transformed data; (Tukey-Kramer’s HSD for α = 0.05).

Considering total PCs, similar distribution patterns between pearling fractions were observed in the three genotypes (Figure 4). F1 to F3 of the genotypes containing husks were those with the highest total PCs with the average content of 4339 μg/g, being on average 3 times higher than the contents in whole flours. On the contrary, F1 of the hull-less genotype was the highest in total PCs.

Most studies into the spatial distribution of PCs in barley have focused on either the total PCs by colorimetric methods [21,22] or just the major phenolic acids [38,39], or have analyzed representative phenolic acids and free flavan-3-ols in a few fractions of previously dehulled cultivars [18,40]. The results obtained in the present study show for the first time the specific distribution of a great variety of phenolic compounds determined by HPLC-MS/MS. These include phenolic acids, flavan-3-ols and flavone glycosides in seven pearling fractions in three food barley genotypes differing in the presence of the husk in the threshed grain.

### 3.4. Anthocyanins

In this work, the anthocyanin content of Kamalamai and Annapurna was negligible, whereas a total of 24 anthocyanins were detected in the purple Hidukusch genotype. These included pelargonidin, cyanidin, peonidin, delphinidin, petunidin and malvinidin conjugates of glucose, acetylglucose, malonylglucose, dimalonylglucose, arabinose, rutinose and dihexose. The total anthocyanin content was 47 μg/g, which was lower than or similar to those detected in other purple barley cultivars [12,41,42]. This indicates a great diversity, probably associated with genetic and environmental factors. Cyanidin dimalonyl glucoside represented 45% of the total anthocyanins followed by cyaniding glucoside, which accounted for 35%. Several studies have shown that anthocyanins are mainly concentrated in the pericarp and some aleurone layers that provide the grain colour [35]. In this study, the distribution of anthocyanins between the barley fractions showed fractions F1 and F2 as the highest (Table 6 and Table S2). The total anthocyanin contents detected in F2 was upon 10 times higher than that in whole flour. Like most phenolic compounds, the anthocyanin concentration decreased progressively from F2 to F7 where minor contents were detected.

### 3.5. Antioxidant Capacity (AC)

Antioxidant capacity is an important integrative parameter for evaluating the potential health benefits of foods. The oxygen radical absorbance capacity (ORAC) values of the free, bound and total phenolic compounds of whole barley flours were in the ranges from 46 to 71, 28 to 58, and 74 to 119 μmol Trolox/g respectively (Table 7).

The contribution of free PCs to the total AC was higher than that of the bound PCs. This suggested that free PCs had excellent AC as determined by the ORAC assay, since they were at much lower concentrations than bound PCs as explained above. The average ORAC values of genotypes containing husks were significantly higher than that of Annapurna. The ORAC values detected in the three barley genotypes were similar or relatively higher than those reported for whole barley flours in previous works [23,43,44,45].

**Figure 5:**
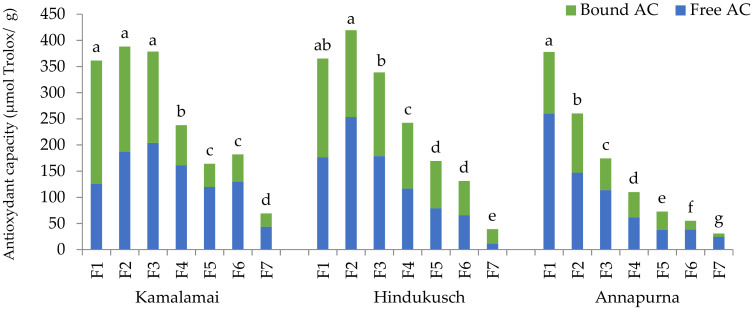
Antioxidant capacity (µmol Trolox/g) detected in seven pearling fractions in three food barley genotypes. Results are presented as the mean; different letters indicate significant differences between pearled fractions of each genotype on log-transformed data; (Tukey-Kramer’s HSD for α = 0.05).

When the ORAC assay was measured in the pearling fractions, the highest values were found in the first three fractions of the genotypes containing husks and F1 of Annapurna (Figure 5). F1 of Kamalamai showed a similar AC to F2 and F3 despite having higher amounts of total PCs. These results should be attributed to the strong antioxidant capacity of free PCs in F2 and F3, which matched the ORAC values of F1. In the case of the purple Hindukusch genotype, the highest AC was detected in F2. This fraction contained the highest amount of free PCs, including anthocyanins, whose AC exceeded that of the components in F1 and F3. Finally, F1 of Annapurna (equivalent to F2 of the genotypes containing husks) showed the highest AC, and this value decreased going toward the inner part of the grain as did free, bound and total PCs. Based on these results, the 0–15% outer fractions of the genotypes containing husks and the 0–5% fraction of Annapurna made significant contributions to the total antioxidant capacity of all whole flours.

### 3.6. Association between Variables

**Figure 6:**
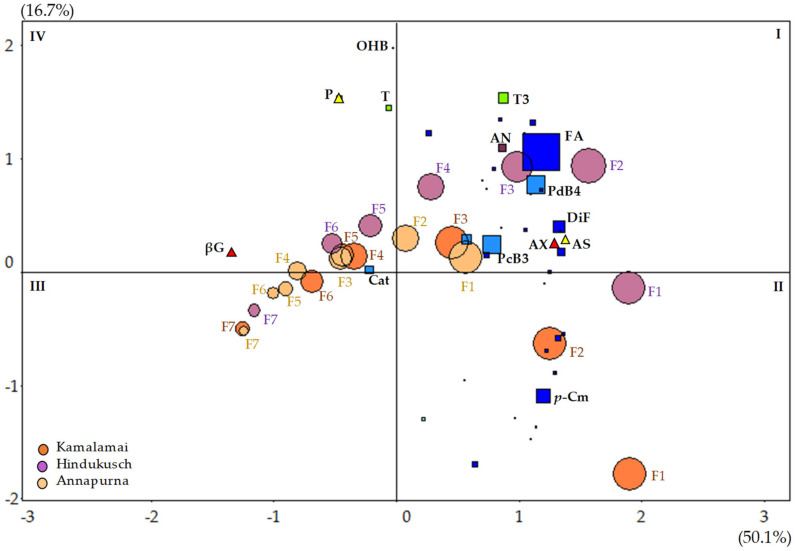
Biplot of the Principal Component Analysis of ash, protein, dietary fiber and 60 bioactive compounds in seven sequential pearling fractions in three food barley genotypes. The size of the squares is proportional to each phenolic compound content; Circle size is proportional to total antioxidant capacity. F: fraction; AS: Ash; P: Protein; βG: β-glucans; AX: arabinoxylans; T: tocopherols; T3: tocotrienols. The following eight main phenolic compounds are also labeled: FA: bound ferulic acid; *p*-Cm: bound *p*-coumaric acid; DiF: bound diferulic acid; Cat: catechins; OHB: *m*- or *o*-hydroxybenzoic acid; PcB3: procyanindin B3, PdB4: prodelfinidin B4; Light blue squares: flavone glycosides; AN: total anthocyanins.

Figure 6 shows the PCA biplot of ash, protein, bioactive compounds and antioxidant capacity in the seven pearling fractions of the three food barley genotypes. The first two principal component axes explained more than two thirds of the total variability in the standardized data from Figure 1, Figure 2 and Figure 3 and and Table 4 and Table 5. The first axis, explaining 50% of the total variation, seemed to be related to the contrast between the bioactive compounds found in the outer fractions (ash, arabinoxylans, phenolic compounds and tocotrienols) and the β-glucans present in the endosperm. The second, explaining 17% of the total, seemed to be related to compounds such as tocopherols, protein and minor phenolic compounds like catechins (Cat) and bound hydroxybenzoic acid (OHB) found in intermediate layers.

The size of the circles for each pearling fractions was proportional to their total antioxidant capacity and clearly descends from fraction F1 to F7 for all genotypes. The distribution pattern was similar for all genotypes, with little differences for the outermost fractions. The size of the blue squares representing the 28 phenolic compounds was proportional to their content within the fractions. Overall, Hindukusch, with the outermost fractions being further away from the origin of coordinates, seemed higher in such bioactive compounds as tocotreinols, prodelphinidin B4 (PdB4) bound ferulic acid (B-F), and anthocyanins (AN) than the other two genotypes. Highlighted in quadrant II, bound *p*-coumaric acid (*p*-Cm) showed higher content in the fractions that mainly contain husks, and in quadrant I the bound ferulic (B-F) and diferulic (B-DiF) acids, and the procyanidin B3 (PcB3) and prodelphinidin B4 (PdB4) present in the outer external fraction.

**Figure 7:**
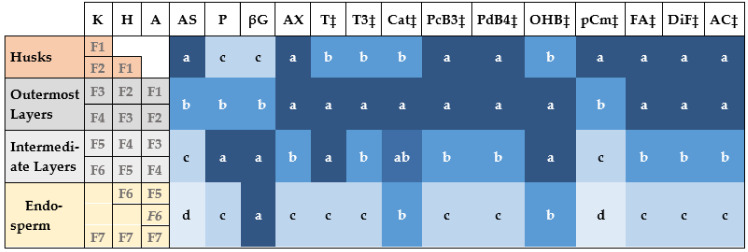
Tukey’s HSD groupings for a number of compounds for the different sections of the grains across three food barley genotypes. The colored left columns represent the different pearling fractions (F1–F7) of the Kamalamai (K), Hindukusch (H) and Annapurna (A) used for the aggregation into four grain sections shown in the first column. A: Ash; P: protein; βG: β-glucans; AX: arabinoxylans; T: tocopherols; T3: tocotrienols; Cat: catechins; PB3: Procyanidin B3; PB4: prodelphinidin B4; OHB: m- or o-hydroxybenzoic acid; pCm: bound p-coumaric acid, FA: bound ferulic acid; DiF: bound diferulic acid; AC: total antioxidant capacity. ‡ Analyses carried out on log-transformed data.

Whereas PCA is an extremely powerful tool for the visualization of multidimensional data, it does not allow for statistical inferences about contents across grain sections. These comparisons are shown in Figure 7, which summarizes the Tukey’s HSD Mean Comparison groupings for four sequential spatial sections of the barley grain, determined by differential aggregation of the seven pearling fraction from each of the three, hulled, partially-hull-less and hull-less food barley genotypes used. The husks had a high content in ash, arabinoxylans, some specific major phenolic compounds such as bound *p*-coumaric, ferulic and diferulic acids, procyanidin B3 and prodelphinidin B4, resulting in a high antioxidant capacity, similar to that of the outermost layers. Current regulations require removal of the inedible husks from hulled barley to be used for human food [46]. However, due to its high content of bioactive compounds, either the whole hulled barley grains or the external husks of food hulled barley genotypes should be considered as a valuable material for animal feeds. Apart from providing the same concentration of bioactive compounds as husks, the outermost layers also had high contents of tocols. Therefore, the outermost layers of naked barley or previously de-husked barley for human food represent the most valuable source of bioactive compounds. The intermediate layers, provide high contents of protein, β-glucans, tocopherols and some phenolic compounds such as catechins and hydroxybenzoic acid. Finally, the endosperm has the highest β-glucans contents and relative high presence of catechins and hydroxybenzoic acid.

In conclusion, high β-glucan food barley genotypes can be an excellent source of not just of dietary fiber but a plethora of phenolic compounds with potential health promoting properties. Whole or lightly pearled grains, as well as their specific pearling fractions, could be used as a diverse source of valuable functional ingredients.

## Figures and Tables

**Table 1:** Ash and protein contents in whole barley flours and pearling fractions.

	Ash (%)			Protein (%)		
	Kamalamai	Hindukush	Annapurna	Kamalamai	Hindukush	Annapurna
Whole flour	1.97 a	1.68 a	1.48 b	14.62 b	15.26 a	14.83 ab
Fractions						
F1	6.64 a	6.52 a	5.16 a	7.77 g	10.54 e	19.97 e
F2	5.60 b	5.75 b	4.74 b	17.88 e	17.17 d	25.63 c
F3	5.00 c	5.86 b	3.81 c	24.18 d	23.10 c	27.88 a
F4	3.93 d	5.09 c	3.23 d	26.72 a	27.57 a	27.34 b
F5	3.17 e	3.94 d	2.26 e	26.11 b	27.34 a	25.33 c
F6	2.50 f	2.76 e	1.77 f	25.21 c	26.81 b	23.11 d
F7	0.87 g	0.76 f	1.13 g	12.80 f	10.54 f	11.93 f
SED	0.04	0.05	0.03	0.13	0.11	0.14

Results are presented as the mean. Means within a column followed by different letters indicate significant differences; (Tukey-Kramer’s HSD for α = 0.05) SED: standard error of the difference between means. F1–7: fraction1–7.

**Table 2:** β-glucans, arabinoxylans and tocols contents in whole barley flours.

	Kamalamai	Hindukusch	Annapurna
β-glucans (g/100 g)	8.41 ± 0.09 b	8.26 ± 0.07 b	9.46 ± 0.10 a
Arabinoxylans (g/100 g)	5.52 ± 0.27 b	6.60 ± 0.44 a	5.52 ± 0.19 b
α-Tocopherol (µg/g)	8.32 ± 0.21 a	7.53 ± 0.22 a	6.22 ± 0.19 b
β-Tocopherol (µg/g)	0.03 ± 0.01 a	0.03 ± 0.01 a	0.03 ± 0.01 a
γ-Tocopherol (µg/g)	1.58 ± 0.00 a	1.08 ± 0.01 c	1.21 ± 0.01 b
δ-Tocopherol (µg/g)	0.03 ± 0.00 a	0.02 ± 0.00 c	0.03 ± 0.00 b
Total Tocopherol (µg/g)	9.97 ± 0.05 a	8.66 ± 0.06 b	7.49 ± 0.05 c
α-Tocotrienol (µg/g)	17.03 ± 0.42 b	20.06 ± 0.90 a	18.84 ± 0.23 a,b
β-Tocotrienol (µg/g)	2.05 ± 0.00 b	3.93 ± 0.15 a	3.61 ± 0.04 a
γ-Tocotrienol (µg/g)	9.06 ± 0.20 a	9.05 ± 0.46 a	5.63 ± 0.03 b
δ-Tocotrienol (µg/g)	2.07 ± 0.02 b	2.72 ± 0.07 a	2.19 ± 0.03 b
Total Tocotrienol (µg/g)	30.21 ± 0.16 b	35.77 ± 0.40 a	30.27 ± 0.08 b
Total Tocols (µg/g)	40.18 ± 0.81 a,b	44.43 ± 0.82 a	37.77 ± 0.81 b

Results are presented as the mean ± standard error of the mean. Means within rows followed by different letters indicate significant differences; (Tukey-Kramer’s HSD for α = 0.05).

**Table 3:** Phenolic compounds contents (µg/g) in whole barley flours.

	Kamalamai	Hindukusch	Annapurna
Flavan-3-ols	500.7 ± 33.2 a	272.3 ± 12.8 b	320.7 ± 14.6 b
Free phenolic acids	81.1 ± 6.1 a	70.6 ± 2.3 a,b	59.6 ± 2.3 b
Free flavones glycosides	18.4 ± 0.5 b	26.0 ± 0.4 a	9.0 ± 0.2 c
Total free	600.2 ± 30.9 a	368.8 ± 14.8 b	389.3 ± 12.2 b
Bound phenolic acids	1092.1 ± 113.4 a,b	1192.6 ± 215.6 a	779.5 ± 32.9 b
Bound flavones glycosides	1.9 ± 0.1 a	1.8 ± 0.0 a	1.8 ± 0.0 a
Total bound	1093.9 ± 113.5 a,b	1194.4 ± 215.6 a	781.3 ± 32.9 b
Total phenols	1694.1 ± 117.4 a	1563.2 ± 211.5 a,b	1038.6 ± 36.7 b

Results are presented as the mean ± standard error of the mean. Means within rows followed by different letters indicate significant differences; (Tukey-Kramer’s HSD for α = 0.05).

**Table 4:** Free phenolic compounds contents in barley pearling fractions.

	Flavan-3-ols (µg/g)				Phenolic Acids (µg/g)												Flavones Glycosides (µg/g)					
	Cat	Cat-g	Pc B3	Pd B4	Gallic	Ferulic	DC diF	*p*-Cm	*p*-OHB	OHB	2,4- diOHB	Vanill	Caff	Syring	Cinna	Ap g	Isosc g	Isosc r	Isoor	Isov g	Isov g-g	Isov r
Kamalamai																						
F1	53 d	102 b	374 c	312 c	35.9 a	27.1 a	1.1 a	25.7 a	21.5 a	10.9 a	16.9 a	78.4 a	11.6 b	23.3 a	12.8 a	13.8 a	13.4 a,b	8.3 a	7.3 a	0.2 d	1.7 a	1.8 a
F2	96 c	173 a	662 a	536 b	37.0 a	20.0 a	1.1 a	17.2 a	16.5 b	10.8 a	14.5 b	48.1 b	12.8 a	17.2 b	11.5 b	10.0 b	14.7 a	8.1 a	7.1 a,b	0.2 c,d	1.7 a	1.1 a,b
F3	125 a,b	194 a	740 a	644 a	39.8 a	12.8 b	-	6.6 b	12.8 c	-	-	14.6 c	12.6 a	10.6 c	-	3.4 c	10.4 d,e	7.2 b	6.6 b	0.4 b,c	0.3 b	0.7 b,c
F4	143 a	125 b	434 b	322 c	38.8 a	7.2 c	-	3.5 b,c	11.3 d	-	-	8.3 d	-	8.3 d	-	1.4 d	11.4 c,d	7.1 b	6.7 a,b	0.7 a	-	0.4 c
F5	121 a,b	76 c	264 d	192 d	37.4 a	7.0 c,d	-	3.1 b,c	11.1 d	-	-	8.6 d	-	8.3 d	-	1.0 e	12.8 b,c	7.0 b	6.9 a,b	0.7 a	-	0.4 c
F6	104 b,c	56 d	202 e	134 e	28.3 b	5.3 d,e	-	2.7 c	10.9 d	-	-	7.0 d	-	7.5 d	-	0.7 f	12.3 b,c	6.9 b	7.0 a,b	0.5 a,b	-	0.4 c
F7	73 d	19 e	61 f	36 f	18.0 c	4.1 e	-	0.4 d	1.5 e	-	-	2.5 e	-	1.1 e	-	0.1 g	9.5 e	1.0 c	1.0 c	0.1 e	-	0.1 d
SED	0.1	0.1	0.1	0.1	0.1	0.1	0.1	0.3	0.03	0.1	0.4	0.1	0.2	0.1	0.1	0.1	0.04	0.02	0.03	0.2	0.1	0.2
Hindukusch																						
F1	34 c	53 b	227 b	385 b	24.1 a,b	24.6 a	1.9 a	14.2 a	21.8 a	15.5	40.6 a	58.4 a	11.7 a	15.0 a	-	11.3 a	8.3 b	9.1 a	7.7 a	1.5 a	-	2.2 a
F2	48 a,b	100 a	359 a	612 a	27.8 a,b	14.3 b	1.1 b	5.9 b	18.8 b	-	31.3 b	44.7 b	10.8 b	11.4 b	-	7.2 b	8.4 b	9.1 a	7.4 a,b	2.1 a	-	2.0 a,b
F3	58 a	101 a	349 a	544 a	29.5 a,b	10.2 c	0.7 c	3.7 b,c	16.5 c	-	21.7 c	36.2 c	10.5 b	9.4 c	-	4.3 c	8.3 b	8.0 b	7.0 b,c	1.5 a	-	1.3 b,c
F4	46 b	56 b	235 b	316 c	31.1 a	7.8 d	-	3.0 c,d	15.3 d	-	15.9 d	28.8 d	-	8.4 c,d	-	2.1 d	7.5 c	7.5 b,c	6.9 c	0.8 b	-	0.9 c,d
F5	33 c	24 c	107 c	129 d	32.6 a	6.2 d	-	1.9 d,e	14.1 e	-	14.1 d	23.5 e	-	7.9 d	-	1.2 e	8.7 b	7.3 c	6.9 c	0.6 b,c	-	0.7 d
F6	27 c	14 d	49 d	48 e	20.5 b,c	4.4 e	-	1.1 e	13.1 e	-	1.9 e	17.2 f	-	7.6 d	-	0.6 f	9.5 a	7.1 c	6.9 c	0.3 c	-	0.3 e
F7	16 d	2 d	13 e	8 f	16.1 c	3.5 f	-	-	10.5 f	-	-	5.9 g	-	0.1 e	-	0.1 g	8.3 b	1.0 d	1.0 d	0.1 d	-	0.03 f
SED	0.1	0.1	0.03	0.1	0.1	0.1	0.1	0.2	0.02	0.1	0.1	0.1	0.2	0.04		0.1	0.03	0.02	0.02	0.2		0.2
Annapurna																						
F1	91 b	349 a	927 a	554 a	25.4 a	11.9 a	-	4.5 a	12.4 a	-	-	25.8 a	11.3 a	11.7 a	12.1 a	3.7 a	10.7 a	8.5 a	-	0.2 b,c	-	1.0 a
F2	85 c	245 a	601 b	339 a,b	24.1 a	8.1 b	-	2.2 b	10.9 b	-	-	16.9 a,b	10.3 b	9.2 b	11.4 a,b	1.4 b	8.7 d	7.2 b	-	0.3 a,b	-	0.5 b
F3	91 b,c	139 b	302 c	164 b	22.3 a	6.1 b,c	-	1.8 b,c	10.9 b	-	-	12.5 b	-	7.9 c	10.9 b	0.5 c	9.0 c,d	7.0 b	-	0.3 a,b	-	0.2 c
F4	105 a	79 c	156 d	71 c	21.3 a	4.7 c,d,e	-	1.8 b,c	10.7 b	-	-	6.5 c	-	7.3 c	-	0.4 c,d	9.7 b,c	-	-	0.4 a,b	-	0.2 c
F5	90 b,c	53 c	108 e	49 c	21.0 a	5.2 c,d	-	1.6 c	10.2 b,c	-	-	6.4 c	-	7.1 c	-	0.4 c,d	11.3 a	-	-	0.5 a	-	0.2 c
F6	89 b,c	29 d	55 f	20 d	22.2 a,b	3.6 e	-	-	10.3 b,c	-	-	4.3 c	-	7.1 c	-	0.2 d	10.6 a,b	-	-	0.1 c	-	-
F7	51 d	15 e	30 g	15 d	16.4 b	4.2 d,e	-	-	9.8 c	-	-	2.2 d	-	1.0 d	-	-	7.7 e	-	-	-	-	-
SED	0.1	0.1	0.1	0.2	0.1	0.1		0.1	0.02			0.1	0.1	0.03	0.3	0.2	0.03	0.01		0.2		0.1

Results are presented as the mean. Means within a column followed by different letters indicate significant differences on log-transformed data; (Tukey-Kramer’s HSD for α = 0.05). SED: standard error of the difference between means. Cat: Catechin, Cat-g: Catechin-glucoside, Pc B3: Procyanidin B3, Pd B4: Prodelphinidin B4, DCDiF: Decarboxylated diferulic acid, *p*-Cm: *p*-coumaric acid, *p*-OHB: *p*-hydroxybenzoic acid, OHB: *m*- or *o*-hydroxybenzoic acid, 2,4-diOHB: 2,4-dihydroxyenzoic acid, Vanill: Vanillic acid, Caff: Caffeic acid, Syring: Syringic acid, Cinna: Cinnamic acid, Ag g: Apigenin 6-C-arabinoside 8-C-glucoside, Isosc g: Isoscoparin 7-glucoside, Isosc r: Isoscoparin 7-rutinoside, Isoor: Isoorientin, Isov g: Isovitexin 7-glucoside, Isov g-g: Isovitexin 7-(6’’’-sinapoylglucoside) 4’-glucoside, Isov r: Isovitexin 7-rutinoside.—Not detected.

**Table 5:** Bound phenolic compounds contents (µg/g) in barley pearling fractions.

	Phenolic Acids (µg/g)																Flavones Glycosides (µg/g)			
	Ferulic	isoF	DiF	TriF	DC DiF	THF DiF	*p*-Cm	*m*-Cm	*p*-OHB	OHB	2,4-diOHB	Vanillic	Caffeic	Syringic	Sinapic	Cinna	Ag g	Isosc-g	Isosc r	Isov g
Kamalamai																				
F1	1367 a	17 b,c	330 a	21.2 a	34.3 a	5.5 a	978 a	21.3 a	22.8 a	3.0 c	3.5 b,c	117 a	5.1 c	19.6 a	14.1 a,b	3.8 d	1.6 a	1.7 d	-	0.05 b
F2	1260 a	15 b,c	165 b	15.8 a	18.2 b	1.9 b	523 b	8.1 b	15.3 b	4.6 a,b	4.2 b	78 b	7.2 b	15.9 a	20.3 a	5.5 a	1.1 a	2.4 a	-	0.09 a
F3	1184 a	15 b,c	151 b	31.7 a	3.0 c	-	182 c	3.5 c	11.1 c	4.9 a,b	6.0 a	44 c	16.1 a	9.4 b	21.1 a	5.9 a	0.2 b	2.1 b	-	-
F4	804 b	20 b	69 c	3.8 b	1.1 d	-	65 d	2.2 c,d	6.8 d	4.8 a,b	2.8 c	18 d	3.9 c,d	4.9 c	11.2 b,c	4.5 b	0.1 c	2.0 b,c	-	-
F5	833 b	32 a	70 c	2.2 b	0.9 d,e	-	53 d,e	3.4 c	6.6 d	5.4 a	-	17 d	3.8 c,d	4.4 c	10.0 b,c	4.4 b,c	0.1 c	2.1 b,c	-	-
F6	575 c	14 c	46 d	2.5 b	0.7 e	-	38 e	1.6 d	5.1 e	4.2 b	-	11 e	2.8 d	3.3 d	7.1 c	3.9 c,d	0.1 c	1.9 c	-	-
F7	206 d	6 d	17 e	1.8 b	0.1 f	-	14 f	0.9 e	3.3 f	4.3 b	-	4 f	0.4 e	2.1 e	1.1 d	3.1 e	0.01 d	0.3 e	-	-
SED	0.1	0.1	0.1	0.3	0.1	0.1	0.1	0.2	0.1	0.3	0.3	0.1	0.1	0.1	0.2	0.04	0.3	0.03		0.01
Hindukusch																				
F1	2030 a	16 b,c	342 a	29.5 a	18.7 a	1.9 a	939 a	12.1 a	45.8 a	4.7 a	37.3 a,b	128 a	5.2 b,c	14.7 a	44.1 a,b	5.3 b	0.8 a	2.0 a	1.7 a	0.2 a
F2	2237 a	20 a,b	374 a	26.2 a	9.2 b	0.6 b	355 b	3.7 b	48.0 a	5.3 a	77.5 a	135 a	6.8 a,b	11.5 a,b	57.4 a	6.3 a	0.3 b	2.0 a,b	1.7 a	0.1 b
F3	1968 a	25 a,b	309 a	23.1 a	5.8 b	-	154 c	1.7 c	33.3 b	5.3 a	63.9 a	99 a,b	8.7 a	10.3 b,c	47.9 a,b	5.6 a,b	0.2 b	1.8 b,c	1.6 a	0.1 b
F4	1643 a	30 a	204 b	10.1 b	3.0 c	-	111 c	1.5 c	22.5 c	5.7 a	26.3 b	69 b	4.4 c	8.4 c	32.1 b	5.0 b	0.1 c	1.7 c	1.6 a	0.1 b
F5	1130 b	24 a,b	114 c	6.7 b,c	1.9 c,d	-	53 d	1.1 c	13.8 d	5.6 a	7.4 c	41 c	3.1 d	5.7 d	17.1 c	4.2 c	0.1 c	1.8 b,c	1.1 a	0.1 b
F6	840 b	25 a,b	74 d	4.2 c	1.3 d	-	35 d	1.3 c	10.1 e	5.3 a	4.2 c	29 c	2.8 d	4.4 d	12.2 c	3.7 c	0.1 c	1.9 a,b,c	1.6 a	0.04 c
F7	353 c	10 c	24 e	1.2 c	0.2 e	-	9 e	0.1 d	5.3 f	5.4 a	3.3 c	9 d	0.4 e	2.4 e	1.1 d	3.2 d	-	0.3 d	-	-
SED	0.1	0.2	0.1	0.2	0.7	0.1	0.2	0.2	0.1	0.4	0.2	0.1	0.1	0.1	0.2	0.04	0.2	0.03	0.01	0.2
Annapurna																				
F1	1850 a	15 b,c	262 a	28.5 a	5.2 a	0.30	169 a	1.7 a	11.1 a	4.6 a,b	-	43 a	5.9 a,b	10.2 a	42.0 a	6.3 a	0.2 a	2.3 a	-	-
F2	1697 a	16 b,c	179 b	13.3 b	1.6 b	-	78 b	0.8 b	8.7 b	4.9 a	-	26 b	11.7 a	7.2 b	27.9 b	5.1 b	0.1 b	2.2 a,b	-	-
F3	1323 b	19 a,b	109 c	3.8 c	0.9 c	-	37 c	0.6 b,c	6.7 c	4.6 a,b	-	16 c	3.6 b	5.2 c	15.4 c	4.4 b	-	2.1 b,c	-	-
F4	968 c	25 a	69 d	3.1 c,d	1.0 c	-	21 d	0.5 b,c	4.9 d	4.3 b,c	-	9 d	2.8 b	3.5 d	8.3 d	3.5 c	-	2.0 c,d	-	-
F5	809 d	20 a,b	58 d	2.1 d,e	2.3 b	-	20 d	0.5 b,c	4.1 e	4.0 c	-	7 d,e	2.7 b	2.8 e	5.4 e	3.4 c,d	-	1.9 d	-	-
F6	623 e	14 c	42 e	2.3 d	2.1 b	-	15 d	0.5 b,c	3.9 e	4.3 b,c	-	6 e	2.6 b	2.5 e	4.1 e	3.3 c,d	-	1.9 d	-	-
F7	259 f	6 d	18 f	1.4 e	0.1 d	-	7 e	0.4 c	3.1 f	4.1 c	-	3 f	-	1.9 f	-	2.9 d	-	-	-	-
SED	0.1	0.1	0.1	0.1	0.1	0.0	0.1	0.2	0.04	0.1		0.1	0.3	0.1	0.1	0.1	0.1	0.02		

Results are presented as the mean. Means within a column followed by different letters indicate significant differences on log-transformed data; (Tukey-Kramer’s HSD for α = 0.05). SED: standard error of the difference between means. iso-F: isoferulic, DiF: Diferulic acid, TriF: Triferulic acid, DC DiF: Decarboxylated diferulic acid, DiF THF: Diferulic tetrahydrofuran, *p*-Cm: *p*-Coumaric acid, *m*-Cm: *m*-Coumaric acid, *p*-OHB: *p*--hydroxybenzoic acid, OHB: *m*- or *o*-hydroxybenzoic acid, 2,4DiOHB: 2,4-dihydroxyenzoic acid, Cinna: Cinnamic acid, Ag-g: Apigenin 6-C-arabinoside 8-C-glucoside, Isosc g: Isoscoparin 7-glucoside, Isosc r: Isoscoparin 7-rutinoside, Isov g: Isovitexin 7-glucoside.—Not detected.

**Table 6:** Anthocyanins contents (µg/g) in barley pearling fractions.

	Pelagonidins	Cyanidins	Peonidins	Delphinidins	Petunidins	Malvinidins	Total Anthocyanins
Hindukusch							
F1	41.0 a	285.3 a	5.87 b	17.8 a	3.42 a	1.15 a	354.5 a
F2	50.0 a	366.3 a	21.59 a	20.3 a	2.01 a	1.29 a	461.2 a
F3	25.0 b	188.6 b	0.45 b,c	12.6 b	1.50 a,b	1.06 a	229.2 b
F4	9.6 c	72.5 c	0.25 b,c	6.4 c	0.89 a,b	0.61 a,b	90.2 c
F5	4.6 d	32.8 d	0.17 b,c,d	3.4 d	0.59 b,c	0.40 a,b,c	42.0 d
F6	2.7 e	19.3 e	0.10 c,d	2.1 e	0.20 c	0.30 b,c	24.7 e
F7	1.1 f	7.0 f	0.02 d	0.7 f	0.04 d	0.14 c	9.0 f
SED	0.1	0.1	0.7	0.1	0.3	0.3	0.1

Results are presented as the mean. Means within a column followed by different letters indicate significant differences on log-transformed data; (Tukey-Kramer’s HSD for α = 0.05). SED: standard error of the difference between means.

**Table 7:** Antioxidant Capacity (µmol Trolox/g) of whole barley flours.

	Kamalamai	Hindukusch	Annapurna
Free AC	70.7 ± 1.3 a	61.9 ± 0.2 b	45.6 ± 0.9 c
Bound AC	39.5 ± 2.4 b	57.4 ± 3.3 a	28.1 ± 0.9 c
Total AC	110.2 ± 1.1 a	119.3 ± 3.5 a	74.0 ± 1.8 b

Results are presented as the mean ± standard error of the mean. Means within a rows followed by different letters indicate significant differences; (Tukey-Kramer’s HSD for α = 0.05).

## Data Availability

Data will be available at https://dataverse.csuc.cat/dataverse/Agrotecnio, accessed on 30 October 2020.

## Supplementary Materials

The following are available online at https://www.mdpi.com/2304-8158/10/3/565/s1, Table S1: Tocopherol and Tocotrienol contents in pearling fractions of three barley genotypes; Table S2: Anthocyanins contents (µg/g) in the pearling fractions of the partially-hull-less and purple genotype.
