# Supplementary material for: Bioactive Compounds and Antioxidant Capacity in Pearling Fractions of Hulled, Partially Hull-Less and Hull-Less Food Barley Genotypes
Source: Foods. 2021 Mar 9;10(3):565. doi: 10.3390/foods10030565 (PMC8001832; doi:10.3390/foods10030565)
Supplement: Supplementary file 1 [file foods-10-00565-s001.pdf]

foods

MDPI

Supplementary Materials

Bioactive compounds and antioxidant capacity in pearling fractions of hulled, partially hull-less and hull-less food barley genotypes

Mariona Martínez-Subirà, María-Paz Romero, Alba Macià, Eva Puig, Ignacio Romagosa and Marian Moralejo *

AGROTECNIO Center, University of Lleida, Av. Rovira Roure 191, 25198, Lleida, Spain ; mariona.martinez@udl.cat (M.M.S.); mariapaz.romero@udl.cat (M.P.R.); eva.puig@udl.cat (E.P.); alba.macia@udl.cat (A.M.); ignacio.romagosa@udl.cat (I.R.); marian.moralejo@udl.cat (M.M.).

* Correspondence: marian.moralejo@udl.cat; Tel.: +34-973702858

Table S1: Tocopherol and tocotrienol contents pearling fraction of three barley genotypes.

	Tocopherols (T) (µg/g)										Tocotrienols (T3) (µg/g)									
	α		β		γ		δ		Total T		α		β		γ		δ		Total T3	
									**Kamalamai**											
F1	6.7	d	0.036	a,b	1.37	d	0.028	c	8.1	d	30.4	f	2.24	f	15.8	f	2.49	e	50.9	f
F2	21.2	c	0.036	a,b	2.40	b	0.050	b	23.7	c	84.1	b	5.52	b	50.8	b	4.70	a	145.1	b
F3	30.1	a	0.041	a	1.01	e	0.081	a	31.2	a	99.8	a	6.65	a	61.5	a	5.37	a	173.3	a
F4	30.0	a	0.038	a,b	1.02	e	0.081	a	31.1	a	73.6	c	4.90	c	42.4	c	4.04	b	125.0	c
F5	26.6	b	0.034	b	1.01	e	0.078	a	27.7	b	49.7	d	3.47	d	27.7	d	3.29	c	84.2	d
F6	25.8	b	0.042	a	4.91	a	0.079	a	30.8	a	35.3	e	2.77	e	19.8	e	2.77	d	60.6	e
F7	5.4	e	0.036	a,b	1.53	c	0.028	c	7.0	e	5.3	g	1.68	g	3.6	g	1.66	e	12.2	g
SED	0.01		0.043		0.02		0.026		0.01		0.02		0.02		0.02		0.04		0.02	
									**Hindukusch**											
F1	8.3	d	0.027	c,d	1.19	d	0.026	a,b	9.5	d	65.1	c	12.23	c	38.1	c	6.02	c	121.4	c
F2	18.0	c	0.026	d	1.17	d	0.024	a,b	19.2	c	104.6	a	19.95	a	61.4	a	9.62	a	195.5	a
F3	27.7	a	0.033	a,b	1.45	c	0.024	a,b	29.2	a,b	108.3	a	20.70	a	62.6	a	10.13	a	201.7	a
F4	29.5	a	0.035	a	1.81	a,b	0.026	a,b	31.4	a	87.9	b	15.90	b	48.2	b	7.92	b	159.9	b
F5	28.1	a	0.036	a	1.96	a	0.025	a,b	30.2	a	57.6	c	9.89	d	30.0	d	5.13	d	102.7	d
F6	23.9	b	0.029	b,c,d	1.67	b	0.033	a	25.7	b	27.7	d	4.91	e	13.2	e	3.13	e	49.0	e
F7	2.8	e	0.030	b,c	1.03	e	0.016	b	3.9	e	3.1	e	2.12	f	2.2	f	1.66	f	9.1	f
SED	0.03		0.033		0.03		0.136		0.03		0.03		0.03		0.03		0.03		0.03	
									**Annapurna**											

F1	19.4	a	0.040	a	2.03	b	0.048	a	21.5	a	114.5	a	14.81	a	35.6	a	5.07	a,b	170.0	a
F2	23.8	a	0.037	a	2.75	a,b	0.055	a	26.6	a	131.2	a	17.92	a	40.1	a	5.73	a	195.0	a
F3	24.8	a	0.042	a	3.20	a	0.058	a	28.1	a	93.4	a	12.28	a,b	25.9	a	4.26	b	135.9	a
F4	23.4	a	0.040	a	3.25	a	0.057	a	26.7	a	58.1	b	7.52	b,c	14.9	b	3.13	c	83.7	b
F5	21.8	a	0.041	a	3.12	a,b	0.058	a	25.0	a	38.8	b	5.20	c	9.6	b	2.57	c	56.2	b
F6	25.3	a	0.046	a	3.46	a	0.061	a	28.3	a	39.2	b	5.78	c	9.4	b	2.55	c	57.0	b
F7	3.8	b	0.038	a	1.16	c	0.021	b	5.1	b	6.3	c	2.18	d	2.1	c	1.61	d	12.2	c
SED	0.11		0.060		0.11		0.088		0.11		0.11		0.13		0.13		0.06		0.11	

Results are presented as the mean. Means within a column followed by different letters indicate significant differences on log-transformed data; (Tukey-Kramer's HSD for $\alpha = 0.05$).
SED: standard error of the difference between means.

Table S2. Anthocyanin contents (μg/ g) in the pearling fractions of the partially-hull-less and purple genotype.

	**F1**		**F2**		**F3**		**F4**		**F5**		**F6**		**F7**		SED
Pelargonidin glucoside	4.55	a	5.48	a	2.77	b	1.14	c	0.58	d	0.31	e	0.11	e	0.08
Pelargonidin acetylglucoside	0.10	a	0.12	a	0.08	a	0.03	b							0.12
Carboxypyrano pelargonidin glu	0.25	ab	0.28	a	0.15	b	0.08	c	0.04	d	0.03	d	0.01	e	0.17
Pelargonidin malonylglucoside	23.18	a	28.40	a	14.68	b	5.55	c	2.59	d	1.58	e	0.59	f	0.10
Pelargonidin dimalonylglucoside	12.63	a	15.37	a	7.35	b	2.83	c	1.38	d	0.80	e	0.35	f	0.13
Cyanidin arabinoside	0.19	a	0.24	a	0.14	b	0.05	c	0.03	d					0.09
Cyanidin glucoside	94.90	a	121.81	a	66.00	b	25.64	c	11.69	d	7.07	e	2.30	f	0.08
Cyanidin acetylglucoside	2.04	a	2.42	a	1.36	b	0.53	c	0.25	d	0.17	e	0.04	f	0.10
Cyanidin malonylglucoside	0.89	a	0.64	a	0.39	b	0.22	c	0.14	c	0.08	d	0.01	e	0.13
Cyanidin dimalonylglucoside	186.08	a	241.24	a	120.71	b	46.04	c	20.68	d	12.00	e	4.70	f	0.09
Peonidin acetylglucoside	0.07	b	0.10	b	0.20	a									0.14
Peonidin malonylglucoside	0.28	a	0.28	a	0.18	a	0.18	a	0.15	a	0.07	b	0.01	c	0.20
Peonidin dimalonylglucoside	5.52	b	21.21	a	0.07	bc	0.07	bc	0.03	bc	0.02	bc	0.01	c	0.99
Delphinidin arabinoside							0.05	a	0.03	a					0.16
Delphinidin glucoside	1.91	a	2.94	a	2.15	a	1.49	a	0.53	b	0.26	b	0.08	c	0.23
Delphinidin malonylglucoside	15.33	a	16.80	a	10.04	b	4.54	c	2.70	d	1.78	e	0.65	f	0.09
Delphinidin dimalonylglucoside	0.59	a	0.57	a	0.39	ab	0.28	b	0.14	c	0.08	d	0.01	e	0.13
Petunidin glucoside	0.44	a	0.46	a	0.40	a	0.23	b	0.17	b					0.05
Petunidin malonylglucoside	2.49	a	1.06	a	0.74	a	0.43	ab	0.26	ab	0.13	b	0.01	c	0.47
Petunidin rutinoside	0.15	ab	0.19	a	0.18	a	0.14	ab	0.09	bc	0.07	c	0.02	d	0.17
Petunidin hexoside hexoside	0.22	a	0.17	ab	0.09	bc	0.05	cd	0.04	d					0.20
Petunidin dimalonylglucoside	0.13	a	0.12	a	0.09	a	0.04	b	0.03	b					0.01
Malvidin malonylglucoside	1.01	a	1.15	a	1.00	a	0.57	a	0.39	ab	0.28	ab	0.12	b	0.44
Malvidin hexoside hexoside	0.03	ab	0.05	a	0.06	a	0.04	ab	0.02	bc	0.02	bc	0.01	c	0.21

Results are presented as mean. Means within a rows followed by different letters indicate significant differences on log-transformed data; (Tukey-Kramer HSD for α= 0.05). SED: standard error of the difference between means.
